# Case of a 30-year-female with systemic lupus erythematosus: a rare clinical image

**DOI:** 10.11604/pamj.2022.42.290.36118

**Published:** 2022-08-18

**Authors:** Switi Jawade, Pratibha Wankhede

**Affiliations:** 1 Nursing Tutor Florence Nightingale Training College of Nursing, Datta Meghe Institute of Medical Science (DU) Sawangi, Wardha, Maharashtra, India,; 2 Department of Community Health Nursing, Smt. Radhikabai Meghe memorial College of Nursing, Datta Meghe Institute of Medical Sciences, Sawangi, Wardha, Maharashtra, India

**Keywords:** Systemic lupus erythematosus, connective tissue disorder, autoimmune disease

## Image in medicine

Systemic lupus erythematosus is a chronic and autoimmune disease that causes inflammation in connective tissue such as cartilage and the lining of blood vessels in which the immune system attacks its tissues. The aetiology factors are unknown but are linked to environmental, genetic, and hormonal factors; they can affect the joints, skin, brain, lungs, kidneys and blood vessels. We here report the case of a 30-year-female patient, who came to the dermatology ward with the complaints of flat red rash across the cheek and bridge of the nose, fluid-filled lesion all over the body, pain and itching, burning associated with the lesion, pedal oedema, oral ulcer, she was apparently alright four days back when she developed fluid-filled lesion which was sudden in onset and gradually progressive, the lesion occurred after ingestion of drug for fever. Initially, blood, electrocardiogram (ECG), chest X-ray investigation done, ultrasound sonography test (USG) done, which was suggestive of mild hepatosplenomegaly with the dilated portal vein and minimal free fluid in the pouch of Douglas, raised cortical echotexture of kidneys, skin biopsy done, and it shows atrophied epidermis, histopathological features suggestive of erythema multiform. The patient was referred to the dermatology and medicine department for further management.

**Figure 1:**
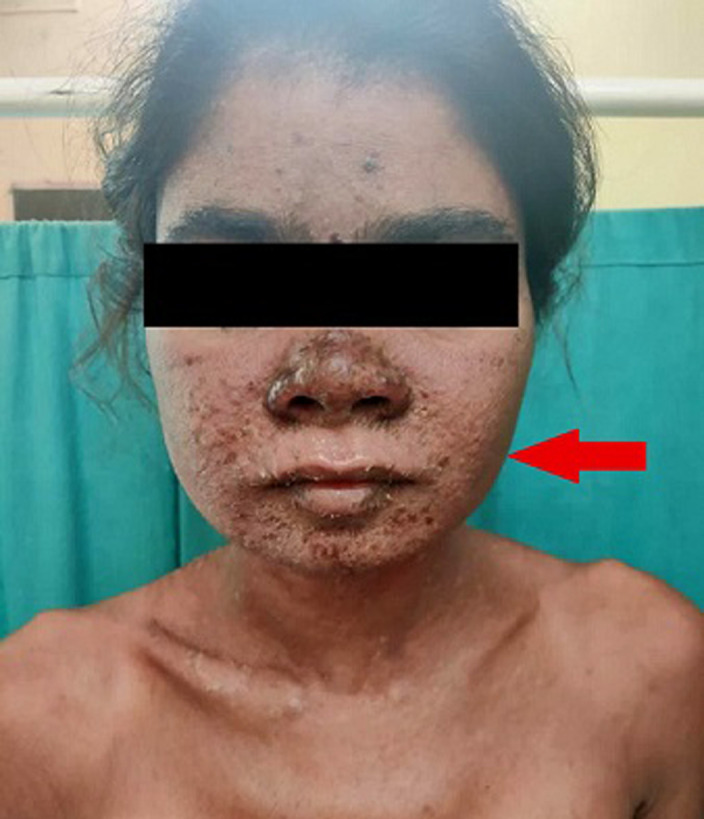
systemic lupus erythematosus

